# *pgxRpi*: an R/bioconductor package for user-friendly access to the Beacon v2 API

**DOI:** 10.1093/bioadv/vbaf172

**Published:** 2025-07-16

**Authors:** Hangjia Zhao, Michael Baudis

**Affiliations:** Department of Molecular Life Sciences, University of Zurich, Zurich 8057, Switzerland; Computational Oncogenomics Group, Swiss Institute of Bioinformatics, Zurich 8057, Switzerland; Department of Molecular Life Sciences, University of Zurich, Zurich 8057, Switzerland; Computational Oncogenomics Group, Swiss Institute of Bioinformatics, Zurich 8057, Switzerland

## Abstract

**Motivation:**

The Beacon v2 specification, established by the Global Alliance for Genomics and Health (GA4GH), consists of a standardized framework and data models for genomic and phenotypic data discovery. By enabling secure, federated data sharing, it fosters interoperability across genomic resources. Progenetix, a Beacon v2 reference implementation, exemplifies its potential for large-scale genomic data integration, offering open access to genomic mutation data across diverse cancer types.

**Results:**

We present *pgxRpi*, an open-source R/Bioconductor package that provides a streamlined interface to the Progenetix Beacon v2 REST API, facilitating efficient and flexible genomic data retrieval. Beyond data access, *pgxRpi* offers integrated visualization and analysis functions, enabling users to explore, interpret, and process queried data effectively. Leveraging the flexibility of the Beacon v2 standard, *pgxRpi* extends beyond Progenetix, supporting interoperable data access across multiple Beacon-enabled resources, thereby enhancing data-driven discovery in genomics.

**Availability and Implementation:**

*pgxRpi* is freely available under the Artistic-2.0 license from Bioconductor (https://doi.org/10.18129/B9.bioc.pgxRpi), with actively maintained source code on GitHub (https://github.com/progenetix/pgxRpi). Comprehensive usage instructions and example workflows are provided in the package vignettes, available at https://github.com/progenetix/pgxRpi/tree/devel/vignettes.

## 1 Introduction

The Beacon v2 specification, adopted as a Global Alliance for Genomics and Health (GA4GH) standard in 2022, provides a standardized framework and data models for secure, federated discovery of genomic and phenotypic data. This addresses critical challenges in data sharing for biomedical research and clinical applications (Rehm *et al.* 2021, Rambla *et al.* 2022). Compared to Beacon v1, which was primarily designed for existence queries on genomic variant collections, returning only binary “Yes” or “No” responses (Fiume *et al.* 2019), Beacon v2 significantly expands its scope. It now supports richer queries, enabling detailed retrieval of both genomic variant data and phenotype information, making it more suitable for clinical and translational research. Recent expansions of the Beacon v2 ecosystem have further enhanced its functionality and accessibility. These include support for deployment strategies for lightweight, local Beacon services to increase adoption and performance (Rueda *et al.* 2022), advanced query composition across data types and metadata layers (Wickramarachchi *et al.* 2023), and integration with Phenopackets v2 and structured phenotype comparisons (Leist *et al.* 2024).

As a reference implementation of Beacon v2 specification, Progenetix, an open-access genomic data resource established in 2001, offers comprehensive mutation profiles of cancer genomes, with a primary focus on copy number variations (CNVs) across diverse human neoplasms (Huang *et al.* 2021). Currently, it hosts approximately 150 000 samples spanning nearly 900 distinct cancer types. These data can be accessed via a user-friendly web graphical user interface (GUI) or programmatically through a representational state transfer (REST) application programming interface (API), fully compliant with the Beacon v2 protocol.

To support simplified and scriptable access to Beacon v2 resources from the R environment, we introduce *pgxRpi*, an R/Bioconductor package designed for querying and retrieving data from Beacon-compliant APIs. While developed with Progenetix as its initial target, *pgxRpi* is designed to be fully generalizable: users can specify any Beacon v2-compatible domain endpoint, allowing access to a wide range of federated resources. In addition to facilitating data access, the package provides powerful visualization and processing functions to support data exploration and downstream analysis. As such, *pgxRpi* represents a practical expansion of the Beacon ecosystem, enabling scalable and reproducible data access workflows directly within the R environment.

## 2 Methods

### 2.1 Retrieving data from the Progenetix Beacon v2 API

**Figure 1.**
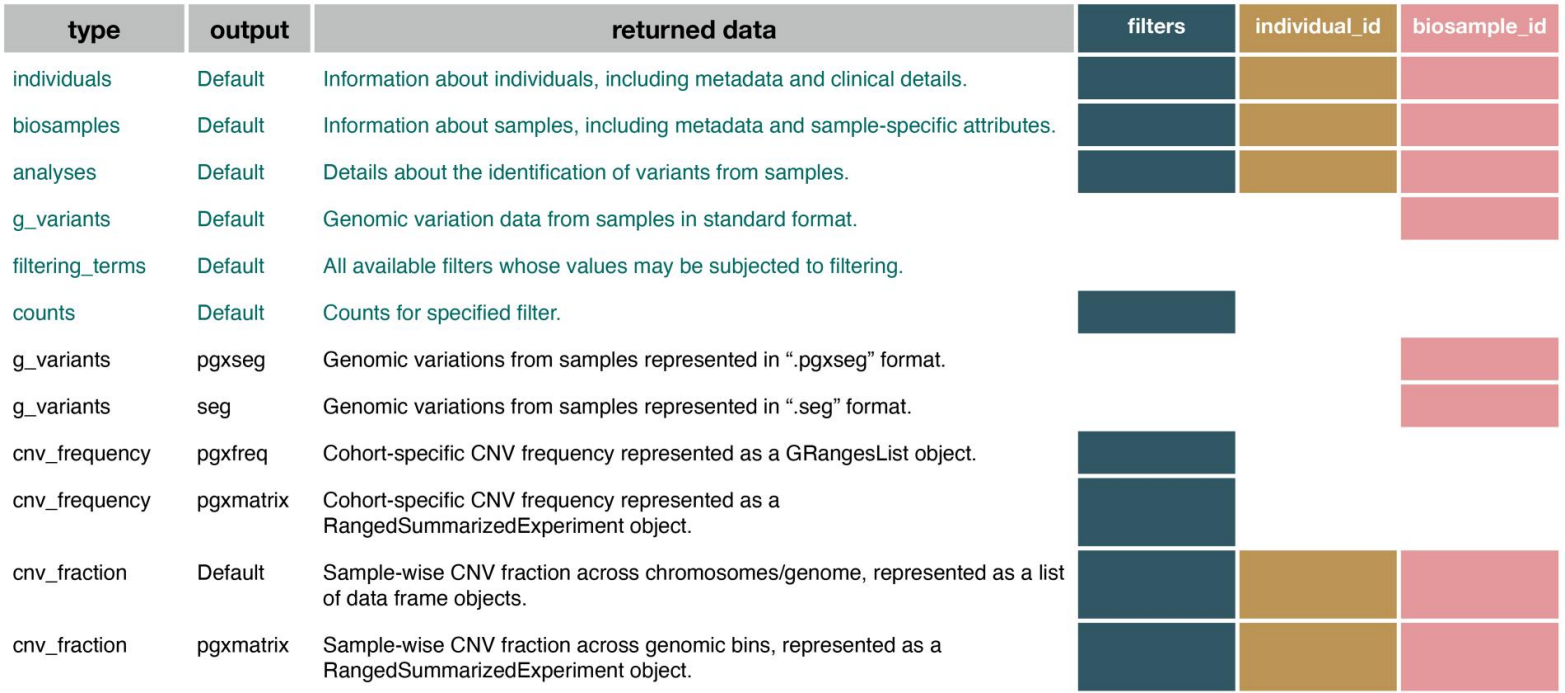
Data retrieval options using the pgxLoader function. The first two columns display the available options for the type and output parameters of pgxLoader, while the third column provides descriptions of the retrieved data. Colored boxes indicate the accessible data types based on the specified search criteria in Progenetix . Data is retrieved through the standard Beacon v2 API (indicated by green text) or the Progenetix-specific "services" API (indicated by black text).

The Progenetix Beacon v2 API, accessible at https://progenetix.org/beacon, facilitates programmatic access to structured cancer genomic data. To streamline data integration in R, we developed the pgxLoader function (Fig. 1), which converts API responses from JSON format into relational tables, enabling more efficient manipulation and analysis. This conversion process is guided by a YAML configuration file located in the inst/config directory of the package, ensuring compliance with the Beacon v2 specification. Details of the JSON flattening strategy used to convert nested API responses into tabular formats are summarized in Supplementary Table S1.

Progenetix data, as defined by the Beacon v2 model, is organized into four main entities: individuals, biosamples, analyses, and genomic variations. These entities correspond to the REST API resources/individuals,/biosamples,/analyses, and/g_variants, respectively. Users can query data for any of these entities through the pgxLoader function by specifying the target entity in the type parameter (e.g., type = “biosamples”). This unified approach eliminates the need to use different endpoints for each data category, simplifying data retrieval (The additional *runs* entity and its /runs endpoint are formally supported by the Progenetix API but provide minimal data content.).

Query specificity can be refined using additional parameters such as biosample_id, individual_id, and filters. These parameters allow users to narrow down results based on criteria defined by the Beacon v2 standard. The biosample_id and individual_id correspond to unique identifiers for biosamples and their associated individuals (*cf.*/biosamples/{id} and/individuals/{id} in REST paths). The filters parameter define rules for selecting records based on specific field values, predominantly referencing bio-ontologies or registered identifiers through the use of compact uniform resource identifiers (CURIEs).

To aid in constructing queries, the Beacon v2 specification includes a /filtering_terms informational endpoint, which lists all queryable fields and their permissible values. *pgxRpi* integrates access to this functionality directly within pgxLoader by setting type = “filtering_term.” Users can search for relevant terms by supplying one or more keyword strings to the filter_pattern argument. When multiple patterns are provided, matching terms for any of the inputs will be returned.

The example below illustrates a typical two-step workflow for query construction. First, pgxLoader is used to search for filter terms related to “male” and “lung adenocarcinoma.” The user then (hypothetically) selects the appropriate National Cancer Institute (NCI) Thesaurus (NCIt) CURIEs from the result and applies them in a second call to retrieve matching biosample records. When multiple filters are supplied, Beacon v2 applies AND logic by default, returning only records that satisfy all specified criteria.

# Step 1: Discover relevant filtering terms

pgxLoader(type = “filtering_terms”,

      filter_pattern = c(“male”, “lung adenocarcinoma”))

# Step 2: Retrieve biosamples using the filters

pgxLoader(type = “biosamples”,

      filters = c(“NCIT:C20197”,”NCIT:C3512”))

In addition to retrieving detailed records, pgxLoader also supports count queries, which return the number of records matching the specified filters. This feature is accessible by setting type=“counts”, providing a quick overview of data availability before executing full queries.

### 2.2 Data retrieval from Beacon v2 compliant resources

The Beacon v2 specification comprises two key components: the framework and the data models. The framework standardizes request and response formats, enabling consistent communication between clients and data providers, while the data models define the structure for representing biological data. This architecture supports data access across diverse genomic resources aligned with the Beacon v2 model.

The pgxLoader function leverages this architecture to query data from any Beacon-compliant resource by specifying the domain and entry_point parameters. For instance, details of individuals with severe COVID-19 infections (C189227) can be retrieved from the Genomic Data Infrastructure (GDI) Spain Node as follows:

pgxLoader(type = “individuals”,

      filters = “NCIT:C189227”,

      domain = “beacon-spain.ega-archive.org”,

      entry_point = “api”)

The Beacon framework ensures standardized communication, although variations in data representation may occur due to differences in data types, access policies, and data granularity across Beacon instances. To address these challenges, *pgxRpi* employs a YAML-based mapping system that harmonizes Beacon v2 responses into a unified format suitable for downstream analysis. This mapping system is optimized for Progenetix but also performs effectively with resources that share structural similarities, such as *cancercelllines.org* (Paloots and Baudis 2024). This resource adopts the same middleware and API stack, built using the *bycon* Python package, and adheres to the Beacon v2 API standard. By leveraging the Beacon v2 framework and data models, *pgxRpi* can efficiently extract and harmonize key data from diverse Beacon-enabled databases, even in the presence of structural differences. This capability not only supports integration of heterogeneous datasets but also ensures the package’s adaptability to future Beacon protocol updates.

Beyond querying individual Beacon instances, *pgxRpi* supports asynchronous multi-domain queries, enabling users to retrieve data from multiple Beacon v2 resources in parallel. The num_cores parameter allows users to control the number of processing cores, accelerating query execution and reducing overall processing time. Performance improvements from multi-core usage are illustrated in Supplementary Fig. S1. The following example shows how to retrieve the number of available records from female (C16576) patients across the GDI Norwegian Node, Progenetix, and *cancercelllines.org*:

pgxLoader(type = “counts”,

      filters = “NCIT:C16576”,

      domain = c(“tryggve.tsd.usit.uio.no”,

           “progenetix.org”,

            “cancercelllines.org”),

      entry_point = c(“beacon/api”, “beacon”, “beacon”))

By enabling parallel data retrieval, this functionality optimizes query efficiency and minimizes computational overhead, making it a powerful tool for large-scale data exploration. Additional implementation details related to parallel querying, partial result handling, and data granularity are summarized in Supplementary Table S1.

### 2.3 Employing the extended functionality of the Progenetix API

The Progenetix Beacon v2 API extends the standard Beacon v2 protocol by providing additional functionality through the ”services” endpoint, enabling specialized access to the full extent of Progenetix data. Services can be accessed in R using the same pgxLoader function (Fig. 1), ensuring a consistent and streamlined approach to querying.

By setting the type parameter, users can retrieve additional data entities beyond the standard Beacon v2 model, including “cnv_frequency” and “cnv_fraction”, which provide pre-calculated CNV features useful for genomic analysis. Additionally, users requiring data formats optimized for analytical workflows can specify the output parameter to obtain results in the desired format. This flexibility ensures that retrieved data aligns with specific analytical needs and facilitates downstream processing.

### 2.4 Data visualization and analysis

*pgxRpi* provides a suite of functions designed to enhance the visualization and analysis of genomic data, allowing users to effectively explore and interpret retrieved datasets. For survival analysis, the pgxMetaplot function, built on the *survminer* package (Kassambara *et al.* 2024), generates Kaplan–Meier survival plots from queried individual data, facilitating comparisons of survival outcomes across different groups. For CNV studies, the pgxFreqplot function, built on the *copynumber* package (Nilsen *et al.* 2012), visualizes CNV frequency data, helping users identify cohort-specific patterns and generate hypotheses. Supplementary Fig. S2 presents example outputs of these visualization functions.

Beyond visualization, *pgxRpi* offers utility functions to streamline data processing. The segtoFreq function computes CNV frequency from segment variant data, supporting both standard “.seg” files and “.pgxseg”—a Progenetix-specific format that integrates CNV variants with metadata. Additionally, pgxSegprocess facilitates the processing of local “.pgxseg” files downloaded from the Progenetix website by extracting segment variants and metadata and organizing them into structured data frames for further analysis. This function also incorporates pgxMetaplot, segtoFreq, and pgxFreqplot, creating a comprehensive toolkit for survival analysis and CNV frequency computation and visualization.

Practical use cases demonstrating these functions are provided in the package vignettes. Examples include workflows that access CNV segment data to compute and visualize cohort-specific CNV patterns, as well as end-to-end pipelines that begin with phenotypic data retrieval and conclude with survival analysis. These examples highlight how *pgxRpi* simplifies Beacon-enabled genomic data analysis by connecting data access with immediate analytical utility.

## 3 Conclusion

The *pgxRpi* package provides a simple, user-friendly interface for accessing and analyzing Beacon v2-compatible genomic data, with Progenetix as a reference implementation. By transforming API responses into structured, analysis-ready formats, *pgxRpi* enhances data accessibility and usability and provides integration with R-based analytical workflows. Typical examples here could be the support of variant calling for CNV analyses through integration of disease-matched CNV frequency data or the comparison of genomic variant calls to reference datasets in clinical genomics applications.

While *pgxRpi* is capable of querying other Beacon v2-compliant resources, the broader realization of cross-resource applications is currently constrained by practical challenges, such as authentication requirements for accessing record-level data, inconsistent ontology usage across resources, and differences in the types of data made available. As a flexible specification, Beacon v2 allows implementations to expose only selected components of the schema depending on local priorities and data availability. While this flexibility supports diverse use cases, it also presents challenges for harmonized, federated analysis. To address these issues, *pgxRpi* includes a unified but adaptable mapping system that aligns heterogeneous metadata and query structures across Beacon implementations. This design improves compatibility across different resources; however, the effectiveness of the mapping can vary depending on the structure and completeness of the underlying data—and is highest for resources that closely resemble the Progenetix data model, for which the package was originally designed.

Importantly, despite current limitations in broad public federation, Beacon v2 and *pgxRpi* offer strong value for data sharing within internal or consortium networks. Institutions and collaborative projects can deploy Beacon v2 services behind secure firewalls, enabling federated querying across departmental or organizational infrastructures. In such controlled environments, *pgxRpi* provides an immediately applicable interface for constructing scalable, private data integration workflows fully compliant with GA4GH standards.

As active contributors to the development and deployment of Beacon v2, we aim to both demonstrate the protocol’s current capabilities and encourage its broader adoption by providing an open, extensible tool that bridges standard-compliant data discovery with practical analysis. This work highlights the broader utility of the Beacon protocol—not only for federated querying but as a foundation for scalable, interoperable genomic data integration. In doing so, *pgxRpi* advances GA4GH’s mission to promote open, standardized, and collaborative research across the global genomics community.

## Supplementary Material

vbaf172_Supplementary_Data
