# Supplementary material for: pgxRpi: an R/bioconductor package for user-friendly access to the Beacon v2 API
Source: Bioinform Adv. 2025 Jul 16;5(1):vbaf172. doi: 10.1093/bioadv/vbaf172 (PMC12321294; doi:10.1093/bioadv/vbaf172)
Supplement: vbaf172_Supplementary_Data [file vbaf172_supplementary_data.pdf]

Supplementary data

pgxRpi: an R/Bioconductor package for user-friendly access to the Beacon v2 API

Hangjia Zhao[1,2*] and Michael Baudis[1,2,*]

[1]Department of Molecular Life Sciences, University of Zurich, Zurich, Switzerland and [2]Computational Oncogenomics Group, Swiss Institute of Bioinformatics, Zurich, Switzerland

Supplementary tables

Aspect	Behavior in *pgxLoader*
Parallel query execution	Variant queries (limited to one domain) are split by sample and executed asynchronously. Multi-domain queries apply to metadata-level queries and are executed in parallel across domains. Results are merged into a unified response.
Synchronous interface	Although sub-queries run in parallel, *pgxLoader* waits for all to complete before returning results, maintaining a synchronous interface from the user's perspective.
Partial results	If all sub-queries return empty, a warning is issued (“No data retrieved”) and NULL is returned.<br>If some requests fail, a warning is issued (“Request failed”) with the domain, entity, and HTTP status code.<br>If some sub-queries succeed but return no matches, a warning is issued (“No matching data”) specifying the domain and entity with no results.
Response granularity	Returns record-level data by default. If type = "counts" is specified, returns summary-level counts matching the query parameters.
Authentication	Only public endpoints are currently supported. Private network use is also supported where no authentication is required.
JSON flattening	Uses a hybrid strategy: predefined mappings for standard fields, and recursive layer-by-layer extraction for flexible or nested fields. Nested keys are concatenated with underscores to form unique column names. Multi-valued entries are collapsed into comma-separated strings.

Supplementary Table S1: *pgxLoader* strategies for Beacon v2 data access.

Supplementary figures

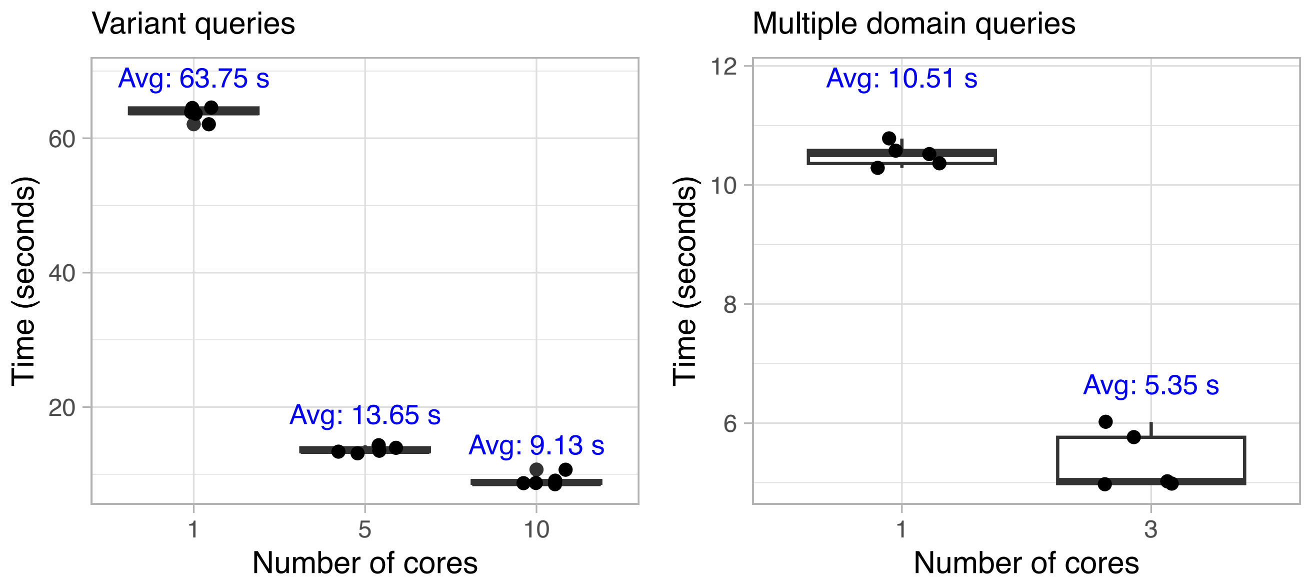

Supplementary Figure S1: Reduction in execution time for variant and multi-domain queries with increased parallelization. The variant query was performed on a set of 50 biosamples, each with approximately 110 CNV segments, retrieved from the Progenetix Beacon v2 API. The multi-domain query corresponds to the example in the main text and includes count queries across three Beacon v2-compliant resources. Each configuration was executed five times under a normal network environment using a MacBook Pro (Apple M1 Max, 10 cores, 64 GB RAM).

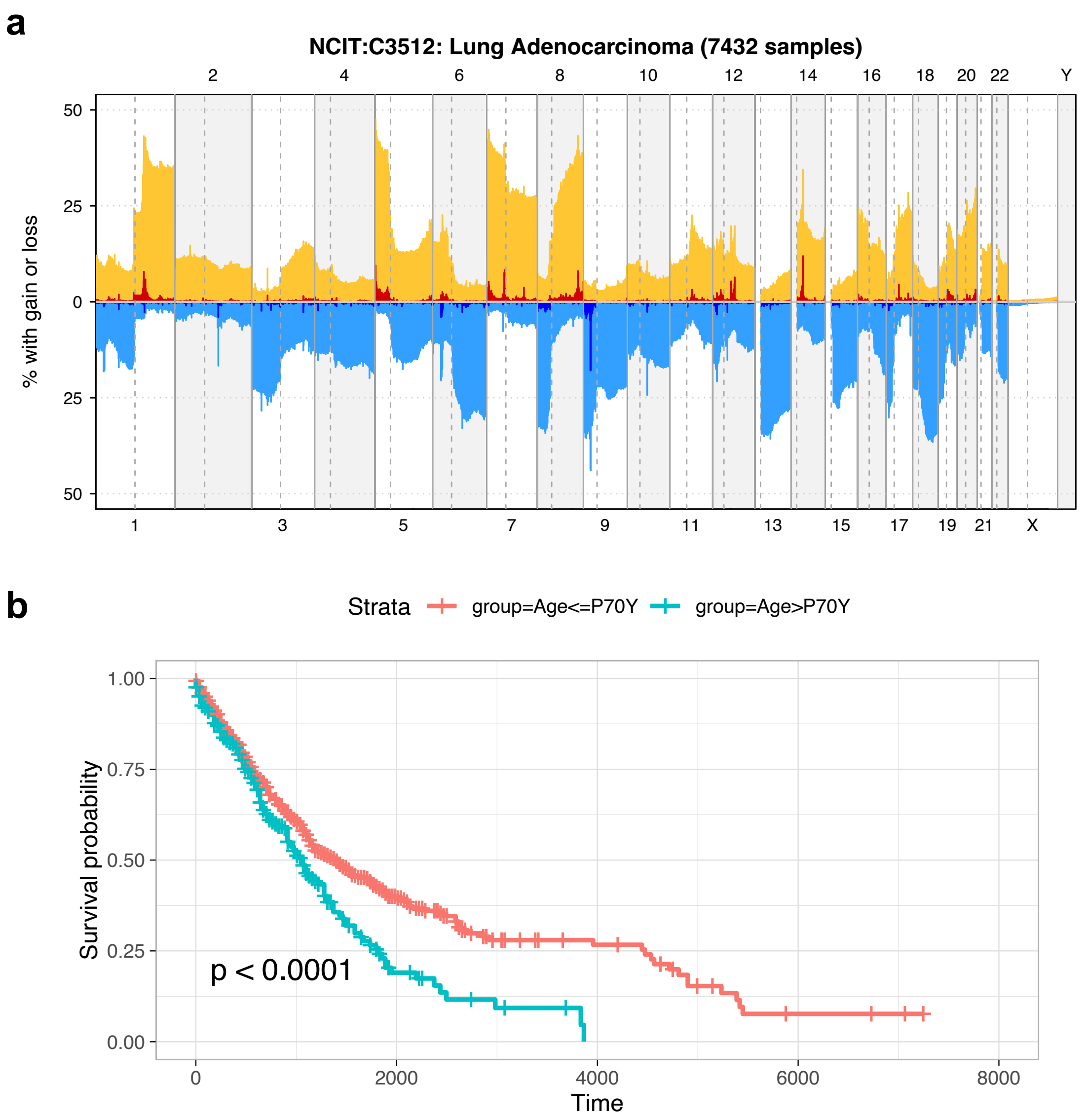

Supplementary Figure S2: Visualization examples. (a) Output of the *pgxFreqplot* function. The Y-axis represents the percentage of lung adenocarcinoma samples in Progenetix with CNAs in 1 MB genomic bins, while the X-axis denotes chromosomal positions. Orange and red indicate low-level and high-level duplications, whereas light blue and dark blue represent low-level and high-level deletions, respectively. (b) Output of the *pgxMetaplot* function. A Kaplan–Meier survival plot comparing survival differences between two age groups based on clinical data from lung adenocarcinoma samples in Progenetix.
